# Cerebrovascular responses to graded exercise in young healthy males and females

**DOI:** 10.14814/phy2.14622

**Published:** 2020-10-28

**Authors:** John D. Ashley, Joe H. Shelley, Jongjoo Sun, Jiwon Song, Jacob A. Trent, Luis D. Ambrosio, Daniel J. Larson, Rebecca D. Larson, Andriy Yabluchanskiy, J. Mikhail Kellawan

**Affiliations:** ^1^ Department of Health and Exercise Science Human Circulation Research Laboratory University of Oklahoma Norman OK USA; ^2^ Department of Health and Exercise Science, Sport, Health, and Exercise Data Analytics Laboratory University of Oklahoma Norman OK USA; ^3^ Department of Health and Exercise Science Body Composition and Physical Performance Research Laboratory University of Oklahoma Norman OK USA; ^4^ Oklahoma Center for Geroscience Department of Biochemistry and Molecular Biology University of Oklahoma Health Sciences Center Oklahoma City OK USA

**Keywords:** brain blood flow, cerebrovascular control, high‐intensity exercise, sex differences, transcranial doppler

## Abstract

Although systemic sex‐specific differences in cardiovascular responses to exercise are well established, the comparison of sex‐specific cerebrovascular responses to exercise has gone under‐investigated especially, during high intensity exercise. Therefore, our purpose was to compare cerebrovascular responses in males and females throughout a graded exercise test (GXT). Twenty‐six participants (13 Females and 13 Males, 24 ± 4 yrs.) completed a GXT on a recumbent cycle ergometer consisting of 3‐min stages. Each sex completed 50W, 75W, 100W stages. Thereafter, power output increased 30W/stage for females and 40W/stage for males until participants were unable to maintain 60‐80 RPM. The final stage completed by the participant was considered maximum workload(*W*_max_). Respiratory gases (End‐tidal CO_2_, EtCO_2_), middle cerebral artery blood velocity (MCAv), heart rate (HR), non‐invasive mean arterial pressure (MAP), cardiac output (CO), and stroke volume (SV) were continuously recorded on a breath‐by‐breath or beat‐by‐beat basis. Cerebral perfusion pressure, CPP = MAP (0. 7,355 distance from heart‐level to doppler probe) and cerebral vascular conductance index, CVCi = MCAv/CPP 100mmHg were calculated. The change from baseline (Δ) in MCAv was similar between the sexes during the GXT (*p* = .091, *ω*_p_^2^ = 0.05). However, ΔCPP (*p* < .001, *ω*_p_^2^ = 0.25) was greater in males at intensities ≥ 80% *W*_max_ and ΔCVCi (*p* = .005, *ω*_p_^2^ = 0.15) was greater in females at 100% *W*_max_. Δ End‐tidal CO_2_ (ΔEtCO_2_) was not different between the sexes during exercise (*p* = .606, *ω*_p_^2^ = −0.03). These data suggest there are sex‐specific differences in cerebrovascular control, and these differences may only be identifiable at high and severe intensity exercise.

## Graphical abstract

We examined cerebrovascular responses to exercise over a wide range of exercise intensities. Our data found that blood velocity responses are not particularly different between the sexes, however, the vascular control is. During high intensity exercise, females vasodilate more than men and men generate more pressure.

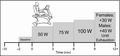

## 1 INTRODUCTION

The brain is highly vascularized and uniquely reliant on the circulation as it is highly sensitive to hydrogen ions, arterial CO_2_ levels (P_a_CO_2_) (Itoh & Suzuki, 2012), and has a lower capacity to store (Brown and Ransom, (2007); Itoh & Suzuki, 2012) or extract large amounts of oxygen (González‐Alonso et al., (2004)), while being particularly sensitive to hyperperfusion (Paulson et al., (1990)). Therefore, exercise presents an interesting challenge to the cerebral vasculature which must simultaneously enhance cerebral blood flow (CBF) to meet changes in metabolic demand, while preventing hyperperfusion of the brain (Paulson et al., 1990). The challenge becomes extraordinary during high‐intensity exercise, where arterial pressure must dramatically increase to support blood flow to working muscles (Mitchell et al., 1983). Thus, in an effort to prevent hyperperfusion, cerebral vessels intrinsically adjust vascular diameter to accommodate changes in arterial pressure, termed cerebral autoregulation (Aaslid et al., 1989; Itoh & Suzuki, 2012; Paulson et al., 1990). Ultimately during exercise, the integration of vasodilator versus vasoconstrictor signals responding to changes in intravascular blood pressure, neuronal oxygen and nutrient consumption, P_a_CO_2_, and ventilation dictate the CBF–exercise intensity relationship (Aaslid et al., 1989; Smith & Ainslie, 2017). The CBF–exercise intensity relationship is biphasic in nature. Such that, CBF increases during low to moderate‐intensity exercise (~ 60%–65% VO_2max_), after which CBF plateaus or reduces back toward baseline at high and vigorous intensities (Smith & Ainslie, 2017).

Historically, female human participants have been underrepresented in the study of human physiology, thereby initiating calls for their inclusion and for human research studies to incorporate research questions aimed at identifying sex‐specific differences (Miller, 2014; Miller et al., 2013). These initiatives have powered some exciting research that has some interesting observations identifying differences in cardiovascular control between the sexes. For example, females tend to have similar blood flow to the periphery (Hart et al., 2011; Parker et al., 2007) with lower blood pressure (Barnes, 2017; Reckelhoff, 2001; Wheatley et al., 2014), cardiac output, and stroke volume (Wheatley et al., 2014) at rest. However, during exercise females tend to have greater skeletal muscle blood flow responses (Kellawan et al., 2015; Parker et al., 2007; Saito et al., 2008), at lower absolute cardiac output, stroke volume (Wheatley et al., 2014), and blood pressure (Kellawan et al., 2015; Parker et al., 2007; Wheatley et al., 2014). This culminates in females having greater vascular conductance (Kellawan et al., 2015; Parker et al., 2007; Saito et al., 2008; Wheatley et al., 2014), a common assessment of vasodilation.

However, it is unclear if these differences exist in the cerebral vasculature during exercise. Only a handful of studies have examined the potential sex differences in CBF and even fewer during exercise. Middle cerebral mean blood velocity (MCAv) tends to be greater (Peltonen et al., 2015; Tegeler et al., 2013; Vriens et al., 1989), or similar (Billinger et al., 2017; Ward et al., 2018), in females compared to males at rest. During moderate‐intensity exercise (Murrell et al., 2013; Ward et al., 2018) and hand‐grip exercise (Joshi & Edgell, 2019) MCAv responses also appear to be similar. However, exercising a single limb or at moderate intensity may not be a great enough perturbance to the system to reveal any differences (Ide et al., 1998). Sex‐specific differences in CBF and cerebral autoregulation have been noted in response to rapid blood pressure changes (Favre & Serrador, 2019) and CO_2_ reactivity (Kastrup et al., 1998). Both of which, are altered during higher intensity exercise.

Therefore, the purpose of this study was to determine if differences exist in the cerebrovascular response to exercise across a wide range of exercise intensities between sexes. We hypothesize that differences in cerebrovascular control between males and females exist at high and vigorous exercise intensities. Specifically, females will have greater MCAv, greater CVCi, and lower CPP during high‐intensity exercise when compared to males as observed in the skeletal muscle circulation (Parker et al., 2007).

## 2 METHODS

### 2.1 Ethical approval

All procedures had ethical approval from the Institutional Review Board at the University of Oklahoma Health Sciences Center (IRB# 10,121). Participants were given a verbal description of all procedures, purposes, and risks involved before providing their informed, written consent. The study conformed to the standards set by the Declaration of Helsinki with the exception of registration in a database.

### 2.2 Subjects

A total of thirty‐two volunteers were recruited for this study. Two subjects were screened out and four subjects had to be removed due to equipment malfunction. Of the twenty‐six subjects remaining, 13 were Females and 13 were Males. All subjects were young (24.2 ± 3.5 years) and free from cardiovascular, metabolic, respiratory, or physical ailments, and none were sedentary (>600 MET/min per week) as determined by a medical history questionnaire and International Physical Activity Questionnaire (iPAQ) long form. All females participating in the study were regularly menstruating or taking oral contraceptives that allowed for regular menstruation. Females were studied within the early follicular phase (1–7 days) of their menstrual cycle or during the placebo phase of their oral contraceptives (Parker et al., 2007; Sims & Heather, 2018).

### 2.3 Protocol

**FIGURE 1:**
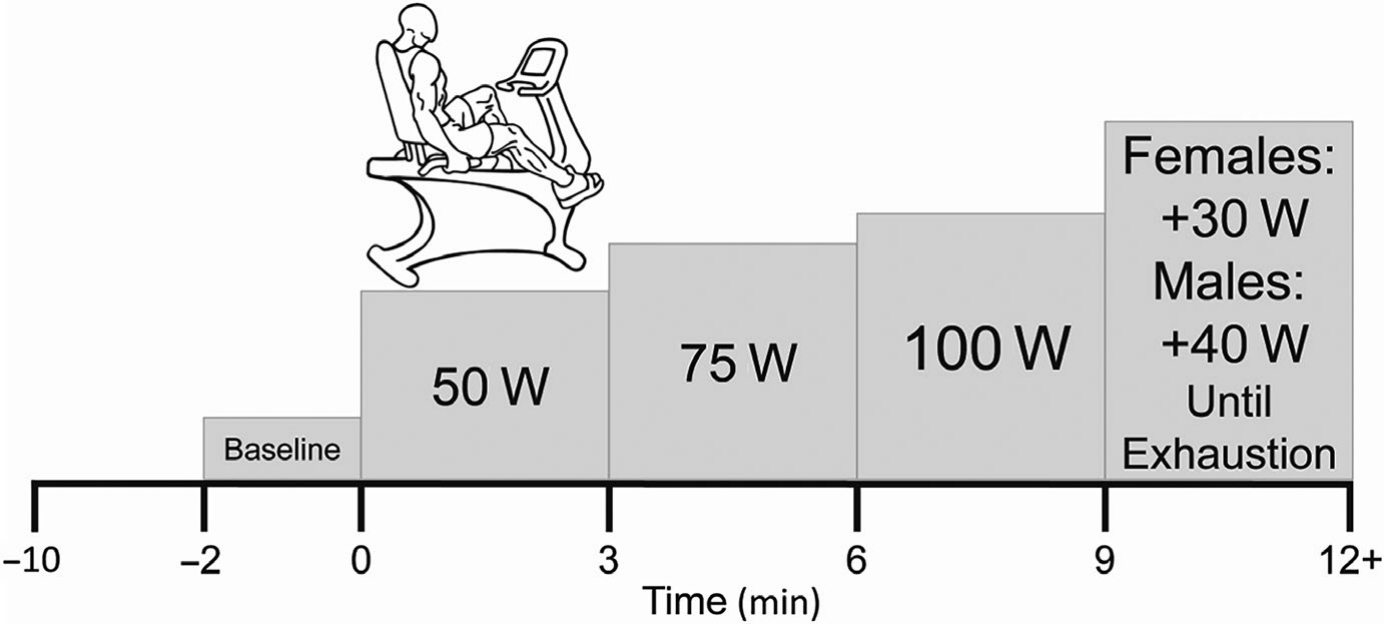
Graded exercise test (GXT) protocol. Subjects were fitted and quality signals for all equipment was ensured prior to test start. At least 2 min of baseline was recorded, followed by 3‐min stages of 50 W, 75 W, then 100 W. Thereon males increased 40 W and females increased 30 W until exhaustion, determined by the inability to maintain 60–80 rpm

All subjects completed one screening and familiarization visit (Visit 1) and one experimental visit (Visit 2). For both visits, subjects arrived at the laboratory ≥ 8 hr fasted, ≥12 hr without caffeine, and ≥ 24 hr without exercise, alcohol, or the use of supplements (e.g., vitamins, other health supplements) and nonsteroidal anti‐inflammatory drugs (e.g., Advil, Aleve). Visit 1, participants provided informed consent, measurements of height (Novel Products, Inc., Rockton, IL), weight (Tanita, Model BWB‐800A), resting blood pressure following ≥ 5 min of supine rest (HEM‐705, Omron) and venous blood samples were collected. Blood samples were analyzed for fasting glucose, triglycerides, cholesterol, LDL, and HDL concentrations (CardioChek PA, Polymer Technology Systems, Inc.) to confirm eligibility. Participants were then familiarized with the testing procedures and a quality transcranial doppler (TCD) signal of the MCA was confirmed. During Visit 2, participants arrived at the laboratory and were fitted with all equipment. Following instrumentation, the participants had five minutes of quiet rest where baseline measurements were recorded. Following baseline, the participants completed a GXT on a recumbent cycle ergometer (Lode Corival cpet, Groningen, The Netherlands) (See Graded Exercise Test below). A recumbent ergometer was used to limit head and upper body movement during large muscle mass exercise testing (Figure 1).

### 2.4 Measurements

Prior to each participant, all equipment was calibrated according to manufacture specifications.

Middle cerebral mean blood flow velocity (MCAv) was continuously measured via TCD during the GXT using bilateral robotic TCD ultrasound probes (2 MHz pulsed‐wave Robotic TCD probe; Neurovision, Multigon Industries). The probes were affixed to an adjustable headband with ultrasound gel applied. Appropriate depth and orientation for optimal vessel insonation were used in accordance with previously published guidelines (Aaslid et al., 1982; Willie et al., 2011). Although these robotic probes are designed to automatically maintain the doppler signal despite head movement, participants were asked to limit the head and upper body movement during the GXT. When possible, both left and right MCA were recorded and data were averaged together as evidence suggests a high level of agreement between left and right MCAv during exercise (Billinger et al., 2017; Ward et al., 2018). Otherwise, the best quality signal from the left or right MCA recording was used.

Heart rate was continuously measured using a wireless ECG (Equivital, EQ02 + *SEM*, Hidalgo). Central cardiovascular variables such as mean arterial blood pressure (MAP), cardiac output (CO), stroke volume (SV), and total peripheral resistance (TPR) were continuously recorded non‐invasively using infrared finger photoplethysmography (ADInstruments, Human NIBP Nano System). Subject's demographic data were entered into the system and within‐software calculations were completed for CO, SV, and TPR. To ensure the integrity of these measurements, the participant's left arm, instrumented with the wrist‐unit of the finger photoplethysmograph, was supported by a sling around the shoulder and the participants were instructed not to squeeze their left hand during testing. These actions were aimed at sustaining a quality signal by preventing the subject from moving or gripping with this limb throughout the GXT.

Pulmonary gasses were assessed using a breath‐by‐breath system (CWE Inc., Gemini End‐Tidal O_2_ and CO_2_ Analyzer; MLT3813H Pneumotach, FE141 Spirometer ADInstruments). This was calibrated prior to each subject using known concentrations of N_2_ and CO_2_ and a 3L flow syringe for ventilation.

### 2.5 Graded Exercise Test (GXT)

Following five minutes of resting baseline measurements, while sitting on the recumbent cycle ergometer, participants completed a GXT that increased intensity in a stepwise fashion every 3‐min requiring a maintenance of 60–80 RPMs. The GXT was designed to have the same first three stages (50, 75, 100 W), after which, power output was increased by 30W for females, and by 40W for males (Figure 1). The GXT was terminated when the participant could not maintain 60–80 RPM, while giving maximum effort and receiving strong verbal encouragement. This GXT design was selected to ensure similar testing times between the sexes (Kim et al., 2016; Parker et al., 2007).

### 2.6 Data acquisition and calculations

Data were acquired at 200 kS/s using PowerLab (PowerLab/16SP ML 880; ADInstruments) and recorded using LabChart software (ADInstruments) on a beat‐by‐beat and breath‐by‐breath basis. Data were then averaged over the last 30 s of each stage completed including baseline. *W*_max_ was recorded as the final stage completed by a participant. If a participant started a stage but could not maintain 60–80 RPM for the full 3‐min, the data from that incomplete stage were disregarded. Cerebral perfusion pressure (CPP) was calculated by adjusting MAP to the hydrostatic column [CPP = MAP (0. 7,355 Ht)] where Ht is the distance (cm) from the TCD probe to the heart (Des, 2012). Cerebral vascular conductance index (CVCi) was calculated by dividing MCAv by CPP (CVCi = MCAv/CPP 100 mmHg). Total vascular conductance (TVC) was calculated by dividing CO by MAP (TVC = CO/MAP 100mmHg). Body surface area (BSA) was estimated using the following formula (BSA = 71.3989 Ht^7437^ Wt^4040^) where Ht is the participant's height (cm) and Wt is the weight (kg) which has been validated for use in both males and females (Yu et al., 2010). Using the BSA, index values of CO and SV were values calculated (CO index, COi = CO/BSA; SV index, SVi = SV/BSA) to normalize for body size (Wheatley et al., 2014). All change values (Δ) were calculated by subtracting the stage value from the baseline value for the variable of interest.

### 2.7 Statistical analysis

Data were analyzed using SAS (SAS 9.1 software. All data are reported as means ± *SD* with significance set at *p* < .05, unless otherwise stated. To answer the hypothesis of this paper, the main outcome variables (MCAv, CPP, CVCi, CO, SV, TVC, and EtCO_2_) were analyzed using an autoregressive, random‐coefficient model (PROC MIXED) fitting a random intercept with a continuous predictor (relative workload, as a percentage of *W*_max_) to determine differences between sexes (Parker et al., 2007). This analysis fits a trend line for the response variable dependent on the relative workload. Due to the uncommon relative workloads for each participant, and the individual differences in response to exercise this statistical approach is more appropriate than traditional repeated measures ANOVA (Parker et al., 2007).

Due to the parabolic nature of several of the variables, a squared term for %*W*_max_ (%W^2^) was used. A pseudo partial omega squared (ω_p_^2^) was calculated using an adapted formula for mixed models in order to determine effect size (Tippey & Longnecker,). Interpretation of ω_p_^2^ is as follows, small ≤ 0.06, moderate > 0.06 and < 0.14, and large ≥ 0.14 (Cohen). For comparison, the model was used to predict values at specific workloads, including at 100%*W*_max_, and compared using an independent *t*‐test with a Bonferroni correction. This analysis was solely for the purpose of post hoc analysis.

Secondary to the hypothesis, an exploratory analysis was completed. A series of panel regressions, a type of longitudinal mixed‐effects model was completed to describe the response of MCAv across exercise intensities. The following equation was used for each of the panel regressions, MCAv = CPP +CO + SV + EtCO_2_ + HR. For this model, CVCi could not be included. Because CVCi was calculated (CVCi = MCAv/CPP) and not directly measured, using it to predict MCAv would introduce several errors. The model equation would read (MCAv = (MCAv/CPP) + CPP…). This would mean CVCi would always be a significant predictor of MCAv. Therefore, in an effort to be overly conservative, CVCi was left out of the regression models. However, the coefficient for CVCi was calculated by taking the inverse of the coefficient for CPP (i.e., CVCi coefficient = 1/CPP coefficient). Regressions were completed for the total response by sex and pooled together. Z‐scores were calculated for each coefficient to determine differences between male and female models (Paternoster et al., 2016). A familywise adjustment was used to determine significance for the difference between coefficients’ Z score, five total parameters therefore *α* = 0.01.

## 3 RESULTS

### 3.1 Demographics

Subject's demographics are presented in Table 1. Unsurprisingly, Males were taller (*p* = .008), weighed more (*p* < .001), had higher systolic blood pressure (*p* = .004), had lower HDL concentration (*p* = .001) and higher estimated BSA (*p* < .001).

**TABLE 1:** Participant demographics

	Males (*n* = 13)	Females (*n* = 13)	*p* value
Age (years)	25 (3)	24 (4)	.517
Height (cm)	178.5 (6.8)	171.5 (5.4)*	.008
Weight (kg)	79.9 (7.6)	64.0 (7.4)*	<0.001
BMI (kg/m^2^)	25.1 (2.1)	21.7 (2.2)*	.001
Systolic BP (mmHg)	120.5 (7.6)	110.4 (8.8)*	.004
Diastolic BP (mmHg)	71.0 (6.6)	71.5 (11.4)	.884
iPAQ Score (MET/min/wk)	3,833 (2,822)	4,871 (3,699)	.441
Total BSA (m^2^)	2.0 (0.1)	1.8 (0.1)*	<.001
Glucose (mg/dL)	94.0 (8.0)	89.9 (7.7)	.190
Triglycerides (mg/dL)	75.1 (18.8)	73.9 (24.8)	.892
HDL (mg/dL)	49.5 (10.0)	65.4 (10.4)*	.001
LDL (mg/dL)	61.3 (23.5)	69.1 (10.8)	.305
*W*_max_ (W)	207.7 (34.2)	153.1 (32.8)*	<.001
VO_2peak_ (L/min)	2.8 (0.7)	2.1 (0.6)	.013

Note

Values are group mean ± *SD*. *n*, number of subjects. BMI, body mass index. BP, blood pressure. iPAQ, international physical activity questionnaire. HDL, high‐density lipoprotein. LDL, low‐density lipoprotein. BSA, body surface area, calculated based off body weight and height. *W*_max_, maximal workload, highest stage completed. VO_2peak_, highest oxygen consumption as measured by pulmonary gases.

* Significant difference between males and females using Student's *t*‐test, *p* < .05.

### 3.2 GTX Performance

Males completed more stages compared to females (5.69 ± 0.85 vs. 4.77 ± 1.09, *p* = .025), achieved a greater peak power during the GXT (207.69 ± 34.19 W vs. 153.08 ± 32.76 W, *p* < .001), and a greater VO_2peak_ (2.8 ± 0.7 vs. 2.1 ± 0.6, *p* = .013). All participants completed the first three stages (50, 75, and 100 W). None of the cerebrovascular response variables were different across the common workloads (Supplemental Table. 1).

### 3.3 Middle cerebral mean blood velocity response to GXT

**FIGURE 2:**
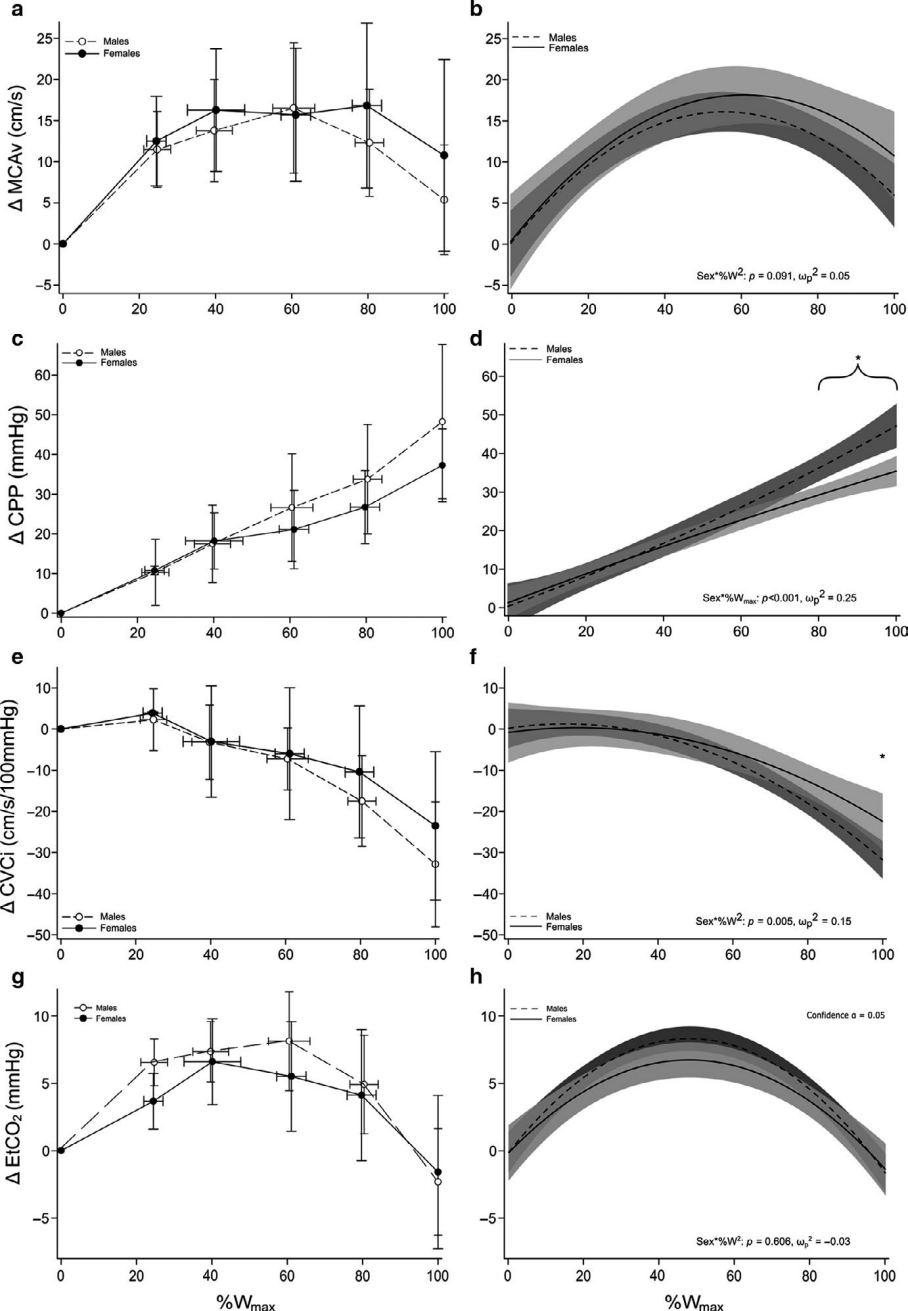
Cerebrovascular and End‐Tidal CO_2_ Responses During Graded Exercise in Young Healthy Males and Females. All values are change (Δ) from baseline over relative work rate (%*W*_max_). Each panel on the left (a, c, e, g) are expressed as group mean ± SD collapsed into common data points of 0%, 20%, 40%, 60%, 80%, and 100% *W*_max._ Each panel on the right (b, d, f, h) is expressed as the regression line of best fit with 95% confidence bands. (a, b) middle cerebral artery flow velocity (MCAv), (c, d) cerebral perfusion pressure (CPP), (e, f) cerebral vascular conductance (CVCi), (g, h) end‐tidal CO_2_ (EtCO_2_). ^*^Significant differences between sexes (graph D at ≥ 80% *W*_max_; graph F at 100% *W*_max_)

Data are displayed graphically in Figure 2a,b. Our model did not reveal significant differences in ΔMCAv (Sex*%W^2^, *F* = 2.89, *p* = .091, *ω*_p_^2^ = 0.05) between males and females. The absolute values also did not reveal any differences (Table 2). Both sexes reached similar MCAv_peak_ values (80.87 ± 13.49 vs. 90.26 ± 19.20 cm/s, *p* = .162) which occurred at similar absolute workloads (121.15 ± 47.75 vs. 100.38 ± 32.69 W, *p* = .208) and relative workloads (57.15 ± 16.15 vs. 65.60 ± 16.60%*W*_max_, *p* = .201, Figure 2a).

**TABLE 2:** Cerebral hemodynamic, central hemodynamic, and pulmonary responses in young healthy males and females during graded exercise

	Relative exercise intensity (%*W*_max_)						*p*‐value		
	Baseline	20%	40%	60%	80%	100%	Sex	Intensity	Interaction
MCAv (cm/s)							.180	<.001	.084
Males	61.66 (2.26)	71.30 (5.69)	76.73 (5.69)	77.95 (5.68)	74.96 (5.69)	67.72 (2.59)			
Females	69.63 (2.43)	79.45 (5.77)	84.92 (5.32)	87.44 (5.75)	85.63 (5.75)	79.94 (2.47)			
CPP (mmHg)							.265	<.001	.002
Males	67.74 (2.88)	77.15 (7.41)	86.56 (7.38)	95.97 (7.38)	105.38 (7.41)	113.08 (3.24)			
Females	75.96 (3.15)	82.82 (7.50)	89.95 (6.90)	96.55 (7.46)	103.42 (7.48)	110.28 (3.07)			
CVCi (cm/s/100mmHg)							.713	<.001	.005
Males	92.73 (3.22)	93.86 (8.13)	91.23 (8.12)	84.85 (8.11)	74.71 (8.12)	61.29 (3.70)			
Females	95.51 (3.47)	97.03 (8.23)	94.86 (7.60)	91.15 (8.21)	83.76 (8.21)	73.39 (3.52)			
EtCO_2_ (mmHg)							.424	<.001	.606
Males	40.24 (1.07)	45.37 (2.71)	47.77 (2.71)	47.42 (2.71)	44.34 (2.71)	38.41 (1.23)			
Females	38.75 (1.15)	43.90 (2.75)	46.19 (2.54)	46.14 (2.74)	43.23 (2.74)	37.62 (1.17)			
TPR (mmHg*s/mL)							.228	<.001	.914
Males	0.93 (0.08)	0.64 (0.20)	0.44 (0.20)	0.35 (0.20)	0.34 (0.20)	0.43 (0.09)			
Females	1.04 (0.08)	0.75 (0.20)	0.55 (0.20)	0.45 (0.20)	0.45 (0.20)	0.54 (0.09)			
TVC (mL/min/100mmHg)							.062	<.001	.002
Males	7.38 (1.27)	12.72 (3.33)	16.43 (3.33)	18.48 (3.33)	18.90 (3.33)	17.34 (1.47)			
Females	4.56 (1.36)	9.72 (3.37)*	12.64 (3.11)*	14.05 (3.36)*	13.22 (3.36)*	10.39 (1.39)*			
CO (L/min)							.043	<.001	<.001
Males	6.09 (1.27)	12.20 (3.59)	16.96 (3.59)	20.36 (3.59)	22.39 (3.59)	22.45 (1.49)			
Females	3.76 (1.34)*	9.57 (3.62)*	13.19 (3.34)*	15.34 (3.61)*	15.28 (3.61)*	13.27 (1.37)*			
COi (L/min/m^2^)							.344	<.001	<.001
Males	2.91 (0.64)	6.13 (1.85)	8.61 (1.85)	10.34 (1.85)	11.33 (1.85)	11.31 (0.76)			
Females	2.39 (0.68)	5.47 (2.87)	7.40 (4.87)*	8.59 (6.87)*	8.63 (8.87)*	7.65 (1.87)*			
SV (mL/beat)							.569	<.001	.002
Males	87.76 (9.48)	127.38 (24.99)	151.54 (24.96)	160.23 (24.95)	153.46 (24.98)	132.21 (10.99)			
Females	81.86 (10.13)	120.15 (25.26)	138.22 (23.30)	142.31 (25.17)	126.18 (25.18)*	91.91 (10.31)*			
Svi (mL/beat/m^2^)							.335	<.001	.002
Males	43.09 (4.95)	64.08 (13.15)	76.84 (13.14)	81.37 (13.13)	77.67 (13.15)	66.54 (5.75)			
Females	48.10 (5.28)	68.38 (13.29)	77.83 (12.26)	80.00 (13.24)	71.34 (13.25)	53.03 (5.37)*			
HR (bpm)							.002	<.001	.007
Males	72.43 (2.57)	94.03 (6.60)	115.46 (6.59)	136.72 (6.59)	157.82 (6.60)	176.54 (2.96)			
Females	84.89 (2.76)*	106.1 (6.68)*	126.4 (6.16)*	145.7 (6.66)	164.1 (6.66)	181.6 (2.80)			
VO_2_ (L/min)									
Males	0.46 (0.10)	0.78 (0.27)	1.18 (0.27)	1.64 (0.27)	2.17 (0.27)	2.70 (0.12)	.156	.002	<.001
Females	0.27 (0.11)	0.58 (0.27)	0.92 (0.27)	1.28 (0.27)*	1.68 (0.27)*	2.11 (0.11)*			

Note

Data are group mean ± SE predicted at common workloads using an autoregressive linear mixed model.

Abbreviations: CO, cardiac output; COi, cardiac output index; CPP, cerebral perfusion pressure; CVCi, cerebral vascular conductance index; EtCO_2_, end‐tidal CO_2_; HR, heart rate; MCAv, middle cerebral artery; SV, stroke volume; SVi, stroke volume index; TPR, total peripheral resistance; TVC, total vascular conductance; VO_2_, ventilated oxygen consumption.

* Significantly Different from Males at the same %*W*_max_, *p* < .05.

### 3.4 Cerebral perfusion pressure response to GXT

Data are displayed graphically in Figure 2c,d. There was a significant interaction (Sex*%*W*_max_) for ΔCPP (*F* = 14.91, *p* < .001, *ω*_p_^2^ = 0.25), Figure 2d. Post hoc analysis of predicted values revealed males had a greater ΔCPP at 80% (*p* = .034), 90% (*p* = .012) and 100% (*p* = .018) *W*_max_. The absolute data also displayed a significant interaction (*p* = .002); however, post hoc analysis did not identify differences at the specific relative workloads examined (Table 2).

### 3.5 Cerebral vascular conductance index response to GXT

Data are displayed graphically in Figure 2e,f. There was a significant interaction (Sex*%W^2^) for ΔCVCi (*F* = 8.18, *p* = .005, *ω*_p_^2^ = 0.15), Figure 2f. Post hoc analysis of predicted values revealed females had greater ΔCVCi at 100% *W*_max_ (*p* = .044). The absolute CVCi also displayed a significant interaction (*p* = .005), however, post hoc analysis did not identify differences at the specific relative workloads examined (Table 2).

### 3.6 Systemic response to GXT

There was a significant interaction (Sex*%*W*_max_) for HR (*F* = 7.40, *p* < .001, *ω*_p_^2^ = 0.11), showing females had greater predicted HR values at Baseline, 20%, and 40% *W*_max_ (Table 2). There was not a significant interaction (Sex*%W^2^) identified for EtCO_2_ (*F* = 0.27, *p* = .606, *ω*_p_^2^ = −0.03), Figure 2g,h and Table 2. There was a significant interaction (Sex*%W^2^) for TVC (*F* = 9.80, *p* = .002, *ω*_p_^2^ = 0.18). Post hoc analysis of predicted values revealed males had greater TVC from 20% *W*_max_ (*p* < .05) to 100% *W*_max_ (*p* < .05). There was a significant interaction (Sex*%W^2^) for CO (*F* = 23.13, *p* < .001, *ω*_p_^2^ = 0.35) and for SV (*F* = 9.78, *p* = .002, *ω*_p_^2^ = 0.18) (Table 2). When adjusted for BSA, the interactions (Sex*%W^2^) remained for COi (*F* = 17.99, *p* < .001, *ω*_p_^2^ = 0.29) and for SVi (*F* = 9.91, *p* = .002, *ω*_p_^2^ = 0.18). Post hoc analysis of predicted values revealed males had greater COi from 60% *W*_max_ (*p* = .004) to 100% *W*_max_ (*p* < .001) and greater Svi at 100% *W*_max_ (*p* = .005) (Table 2).

### 3.7 Explaining cerebral blood flow response

Panel regression results for MCAv with CPP, CO, SV, EtCO_2_, and HR are predictors are presented in Tables 3 and 4. When sexes were pooled together, CPP and EtCO_2_ were significant predictors of MCAv, (*p* = .006 and *p* < .001, respectively), *R*_a_^2^ = 0.703. Data were then split by sexes and separate regressions were conducted and compared. Males exhibited similar results with CPP and EtCO_2_ being significant predictors (*p* = .046 and *p* < .001), *R*_a_^2^ = 0.678, while females had CPP (*p* < .001), EtCO_2_ (*p* < .001), and HR (*p* = .005), *R*_a_^2^ = 0.762. Comparative analysis showed differences between the coefficients for CPP (0.14 ± 0.07 vs. −0.83 ± 0.23, *p* < .001), and EtCO_2_ (1.51 ± 0.15 vs. 10.61 ± 0.96, *p* < .001) for males and females, respectively.

**TABLE 3:** Panel regression data for MCAv response throughout GXT

	CVCi^a^	CPP	CO	SV	EtCO_2_	HR
*Pooled (n = 26, R^2^ = 0.703)* *						
*β* Coefficient	6.77	0.15* (0.05)	−0.11 (0.21)	0.00 (0.03)	1.52* (0.10)	0.05 (0.03)
*p*‐value of coefficient		.006	.599	.867	<.001	.115
*Males Only (n = 13, R^2^ = 0.678)* *						
*β* Coefficient	7.13	0.14* (0.07)	0.31 (0.30)	−0.05 (0.05)	1.51* (0.15)	−0.02 (0.05)
*p*‐value coefficient		.046	.303	.307	<.001	.749
*Females Only (n = 13, R^2^ = 0.762)* *						
*β* Coefficient	−1.20	−0.83* (0.23)	0.06 (0.39)	−0.01 (0.04)	10.61* (0.96)	0.10* (0.04)
*p*‐value coefficient		.001	.872	.906	<.001	.005

Note

Standard errors are reported in parentheses. Each panel contains results from the regression analysis. The first row of each panel is *β* coefficients (SE). The second row of each panel is *p* values associated with the *β* coefficients directly above it. The top panel “Pooled” results from regression analysis with both males and females pooled together. adjusted *R* ^2^ = 0.703, *p* < .001. The middle panel “Males Only” results from regression analysis with only male subjects. adjusted *R* ^2^ = 0.678, *p* < .001. The bottom panel “Females Only” is results from regression analysis with only female subjects. adjusted *R* ^2^ = 0.762, *p* < .001.

^a^ CVCi could not be added to the regression model. Therefore, was calculated by 1/CPP, and does not have an SE or *p* value

* Indicates significance *p* < .05.

**TABLE 4:** Comparison of regressions between sexes

	β coefficients			
Parameters	Males	Females	*Z* Score	*p*
CPP	0.14	−0.83	3.99*	.000
CO	0.31	0.06	0.51	.306
SV	−0.05	−0.01	−0.66	.254
EtCO_2_	1.51	10.61	−9.39*	.000
HR	−0.02	0.10	−1.93	.027

Note

*β* coefficient results from regression analysis, refer to Table 3 for more details. *Z* score was calculated to compare the *β* coefficients from the male and female regression models (Larsen et al., 2008). Familywise adjustment was used to avoid error.

* *Z* score was significant, *α* = 0.01

## 4 DISCUSSION

The purpose of the present study was to determine differences in cerebral vascular control between males and females across exercise intensities during a GXT on a recumbent cycle ergometer. The primary findings of this study were (a) cerebral blood flow velocity response, measured at the MCA, throughout the GXT had a small effect size (*ω*_p_^2^ = 0.05), and the model did not show differences between males and females (Figure 2b), (b) CVCi had a large effect size (*ω*_p_^2^ = 0.15) and was greater in females compared to males at 100% *W*_max_ (Figure 2f), and (c) perfusion pressure, CPP, had a large effect size (*ω*_p_^2^ = 0.25) and was greater in males compared to females at ≥ 80% *W*_max_ (Figure 2d). These data suggest mechanistic differences in the maintenance of CBF between males and females which manifests during high intensities exercise.

### 4.1 MCAv response to GXT

The ΔMCAv response throughout the GXT displayed a parabolic relationship (Figure 2a,b). This type of relationship has been well‐documented (Larsen et al., 2008; Moraine et al., 1993; Smith & Ainslie, 2017; Subudhi et al., 2008, 2011). In agreement with others (Favre & Serrador, 2019; Joshi & Edgell, 2019; Ward et al., 2018), we found no differences in MCAv at baseline (*p* = .182). However, others have noted that females have greater MCAv compared to males at baseline (Peltonen et al., 2015; Tegeler et al., 2013). We also did not find differences in ΔMCAv throughout GXT (Sex * %W^2^ *p* = .091). Though limited, this is in agreement with other studies that have compared CBF between sexes during moderate‐intensity exercise (Murrell et al., 2013; Ward et al., 2018). This suggests males and females supplied similar amounts of blood to active portions of the brain under intense exercise, even though at max, males were doing significantly more work (*p* < .0001). It is important to state TCD does account for any possible changes in vessel diameter; therefore, it measures blood velocity not blood flow (a more in‐depth discussion in Methodological Considerations). Therefore, any statements on blood flow are making assumptions on vessel diameter. We are not aware of any investigations that have confirmed MCA diameter changes over any range of exercise intensities, let alone, differences between the sexes. At rest, MCA diameter has been found to be greater in males compared to females (Muller et al.; Shatri et al., 2017) which is given the same blood velocity would indicate a greater blood flow in males. In contrast, Zarrinkoob et al. completed a large sample size investigation (~94 young and old individuals) using high‐resolution phase‐contrast MRI. They observed similar levels of total brain blood flow between the sexes and greater perfusion in females at rest (Zarrinkoob et al., 2015). When stress such as CO_2_ is introduced, MCA dilation is similar between the sexes (Miller et al., 2019). Similarly, under hypoxic and hypercapnic breathing, Peltonen and colleagues found MCAv response to 90% and 80% SpO_2_ were similar (Δ 5 vs. 8 cm/s and Δ 13 vs. 14 cm/s) and hypercapnia at 10 mmHg above EtCO_2_ baseline was similar (Δ 19 vs. 23 cm/s, males and females, respectively) (Peltonen et al., 2015). These findings are similar to Favre and Serrador who observed no differences in MCAv between sexes when given a hypercapnic gas or hypocapnic breathing (Favre & Serrador, 2019). However, small and large changes in pressure caused by postural shifts did reveal differences in cerebral autoregulation between males and females (Favre & Serrador, 2019). These data suggest potential differences observed in vessel diameter or flow velocity are minimized in the presence of various stresses. The current study measured MCAv responses during graded exercise not at rest. During exercise, CBF must change according to perturbances in perfusion pressure, arterial blood gases, cardiovascular and metabolic changes (Querido & Sheel, 2007). Measured by MRI, CBF is preferentially reduced in the presence of elevated CPP to prevent hyperperfusion (Curtelin et al., 2018). Given males had elevated CPP compared to females (Figure 2d), we suggest the pressure differences subdued any underlying differences in vessel dilation.

### 4.2 CPP response to GXT

Although flow velocity may not have been explicitly different in our study, *how* males and females achieved that similar flow velocity was different. ΔCPP displayed a linear trend with exercise intensity. Males had a higher ΔCPP at intensities of 80% *W*_max_ and greater (Figure 2d). It is well‐documented males have higher systemic and cerebral perfusion pressure at rest and during exercise (Ogawa et al., 1992; Parker et al., 2007; Tegeler et al., 2013). The differences in exercise pressure response seem to be a combination of hormonal, baroreflex resetting, mechano‐, and metaboreflex, and sympathetic outflow, all showing sex differences (Hart et al., 2011; Ives et al., 2013; Jarvis et al., 2011; Katayama et al., 2018; Kim et al., 2012; 2012). For example, Ives and coworkers measured leg blood flow and central hemodynamics during three minutes of passive leg extension in young males and females. Females were found to have a lower cardiac output and blood pressure response to the passive leg movement, indicating an attenuated mechanoreflex in the females (Ives et al., 2013). In studies where metaboreflex activation is isolated via active limb occlusion post‐exercise, females have shown to have a lower muscle sympathetic nerve activity and a blunted blood pressure response compared to males (Ettinger et al., 1996; Jarvis et al., 2011). These data are congruent with observations of lower sympathetic nerve activity and catecholamine release during exercise in females (Gustafson & Kalkhoff, 1982; Katayama et al., 2018). Therefore, the difference in ΔCPP observed in this study (Figure 2d) is most likely due to reduced sympathetic activity associated with an attenuated mechano‐ and metaboreflex response in females. At higher exercise intensities, sympathetic outflow increases (McClain et al., 1994) and would likely accentuate any sex‐related differences. Animal studies have demonstrated increasing MAP increases sympathetic nerve activity resulting in global CBF reduction (Cassaglia et al., 2008; Tuor, 1990). This suggests a link between sympathetic activity and cerebral autoregulation. This link is not widely accepted as several other mechanisms have been shown to play large roles in cerebral autoregulation (Ainslie & Brassard, 2014; Brassard et al., 2017). However, human studies that have altered sympathetic activity show cerebral autoregulation is impaired. Stimulating sympathetic activity results in reduced CBF (Lee et al., 1997), while blunting sympathetic activity increases CBF (Laan et al., 2013), even in the presence of increased MAP (Ogoh et al., 2007). Although the present study did not measure sympathetic outflow, it can be reasoned that at higher exercise intensities sympathetic outflow was its highest. It is likely males had greater sympathetic outflow during the GXT (Ives et al., 2013; Jarvis et al., 2011; Katayama et al., 2018) resulting in greater vasoconstriction. While this would suggest differences in blood velocity, the current findings do not support this (Figure 2b).

### 4.3 CVCi response to GXT

Differences in the vessels’ dilatory response are present in the current study. ΔCVCi displayed a typical curvilinear response (Smith & Ainslie, 2017; Subudhi et al., 2011), decaying below the baseline to a nadir at 100%*W*_max_. Within the context of our experiment, CVCi can be interpreted as the tone of small vessels, downstream from the MCA (site of TCD measurements), in response to changes in blood gases (i.e., EtCO_2_) and pressure autoregulation (i.e., CPP). Interestingly, as exercise intensity increased, males had a greater reduction in ΔCVCi compared to females at 100%*W*_max_ (*p* = .044, Figure 2f). This can be interpreted as females having greater dilation in the cerebral vasculature compared to males. Indeed, it is well‐documented females have greater conductance (i.e., greater dilation) compared to males in the periphery (Gagnon et al., 2013; Kellawan et al., 2015; Parker et al., 2007; Sato et al., 2011; Wheatley et al., 2014). To the best of our knowledge, no investigations have successfully manipulated each of these factors individually during exercise or compared differences in responses between the sexes. However, there have been many investigations that have examined cerebrovascular responses to changes in pressure or arterial CO_2_ in isolation. 4D‐MRI analysis of the major cerebral arteries during hypercapnia has found young males to have a greater cerebral vascular reactivity than young females (Miller et al., 2019). However, this finding is not consistent especially in studies that have measured CBF with TCD (Fan et al., 2019; Madureira et al., 2017; Peltonen et al., 2015). In our study, the ΔEtCO_2_ responses were similar between the sexes as exercise intensity increased (Figure 2h). Therefore, sex‐specific differences observed as exercise intensity increased are likely not driven by changes in EtCO_2_. This statement is supported by studies showing no difference in cerebral reactivity in response to EtCO_2_ changes (Favre & Serrador, 2019; Peltonen et al., 2015). However, differences have been observed between males and females regarding cerebral autoregulation in response to repeated blood pressure oscillations (Labrecque et al., 2019). This finding is not universal (Favre & Serrador, 2019) and could be attributed to variations in study design, protocol, and subject fitness level.

Our data suggest the greater ΔCVCi observed in females is likely due to the cerebral autoregulation of pressure (Figure 2d) during GXT and not due to EtCO_2_ levels (Figure 2h). Further investigation into the underlying mechanisms could include the measurement of venous blood gases for further evaluation of metabolite utilization toward maximal exertion, where we have shown these differences in cerebral autoregulation are at their greatest. This highlights a significant finding in our study, where both sexes maintained a similar cerebral blood flow response (there is a trend for females to have a higher response Table 2) as exercise intensity increased. However, how that response did not achieve statistical significance. Males significantly increased CPP toward maximal exertion whereas females attenuated changes in CPP with less vasoconstriction. This further indicates the mechanisms for maintaining CBF differ between males and females, especially at higher exercise intensities.

### 4.4 Explaining cerebral blood flow response

As an exploratory analysis, we used a panel regression (see Methods section Statistical Analysis) to describe the MCAv response to GXT. From our panel regression analysis, we were able to account for 70.6% (pooled), 67.8% (males) 76.2% (females) separately, of the total variance of MCAv throughout the GXT (Table 3). The goal of the regressions was comparison, not parsimony. Therefore, every model had the same parameters (MCAv = CPP +CO + SV + EtCO_2_ + HR). Doing this allowed for comparison of models and to aid in the explanation of cerebrovascular differences between males and females. Comparing the sex‐specific model β coefficients using a Z score (Paternoster et al., 2016), CPP and EtCO_2_ are significantly different between the sexes. Females reduced MCAv in response to CPP (−0.83 coefficient) compared to males who increased MCAv in response to CPP (0.14 coefficient). Additionally, females and males differentially increased MCAv in response to EtCO_2_ (10.61 vs. 1.51 coefficients). Many studies demonstrate females have a greater MCAv response to P_a_CO_2_ changes (Fan et al., 2019; Kastrup et al., 1998); however, this is not universally accepted (Miller et al., 2019; Peltonen et al., 2015). This analysis is in line with the former, showing females increased MCAv to a greater extent for every change in EtCO_2_ (mmHg), Table 3 and Table 4. These data seem contradictory to the previous discussion. Although this regression analysis showed EtCO_2_ contribution to MCAv response was different between sexes, the MCAv response throughout the GXT was not different (Figure 2b). CPP response was different (Figure 2c), and the contribution of CPP to MCAv response was also different between the sexes (Table 3 and Table 4). Therefore, based on our testing results, we postulate pressure is the main factor driving MCAv response throughout GXT. Important to note, we did not include CVCi in our model, as it would have introduced a mathematical error (see Methods, Statistical Analysis). Therefore, in our effort to be overly conservative, we have likely underestimated the role of CVCi changes and overestimated the role of other variables.

### 4.5 Methodological considerations

We have attempted to control a number of possible confounding variables to the best of our ability. However, several methodological considerations must be acknowledged when interpreting the data from this study. First, all female participants in our study were observed during the early follicular phase of the menstrual cycle to reduce possible cardiovascular hormonal influences (D’Urzo et al., 2018; Kellawan et al., 2015; Krejza et al., 2003; Limberg et al., 2010; Parker et al., 2007). There is evidence of the menstrual cycle altering many physiological responses (Sims & Heather, 2018). Specifically, cerebrovascular resistance during the late follicular is decreased at rest (Krejza et al., 2003). However, under stress, as in this study, the menstrual cycle phase does not appear to affect cerebrovascular control (Favre & Serrador, 2019). It is crucial to note the evidence of menstrual cycle phase effects on cerebral vessels is lacking. Therefore, we took a conservative approach and tested all females in the early follicular phase, reducing the influence of the menstrual cycle phase (Favre & Serrador, 2019; Krejza et al., 2003).

Second, we used TCD to measure blood flow velocity at the MCA which does not account for vessel diameter. Therefore, we cannot accurately measure volumetric blood flow. Recent evidence suggests cerebral vessel diameters can change in response to hand‐grip exercise and changes in arterial gases (Kellawan et al., 2016, 2017; Verbree et al., 2014, 2017). However, it remains unknown if MCA diameter changes during large muscle mass exercise and it can be quite difficult to predict due to the opposing forces of pressure and blood gases changes (Smith & Ainslie, 2017). However, even the best estimates of large cerebral vessel diameter during exercise seem to suggest minimal diameter changes (Giller et al., 2000). Therefore, TCD is still a valid non‐invasive measurement of MCAv that provides high temporal resolution, offering a surrogate for the determination of MCA blood flow during exercise.

Third, this study was completed in young, healthy, relatively active individuals. These participants were of average physical activity level and fitness (iPAQ 3,833 and 4,871 MET/min/wk and VO_2peak_ 2.8 and 2.1 L/min for males and females, respectively) and were free from any metabolic, cardiovascular, or musculoskeletal diseases. The effects of fitness on the present findings are unclear as data on this topic is very limited. Fitness level appears to correspond with increased cerebral hemodynamics in females (Brown et al., 2010) and males (Bailey et al., 2013), limited change (Murrell et al., 2013), or even reduced hemodynamics in females (Labrecque et al., 2019). Thus, it would seem unlikely that examining sex differences within a population with a differing fitness level (e.g., highly trained females compared to highly trained males or sedentary females to sedentary males) to those examined in this investigation would produce differing results and interpretations. Conversely, we would not support these data being generalized to diseased populations. Various diseases have been shown to impair cerebral hemodynamics (Benedictus et al., 2017; Magyar et al., 2001). These diseases affect cerebrovascular control differently and speculation of each of these mechanisms is beyond the scope of this study.

### 4.6 Conclusion

To our knowledge, this study is the first to compare cerebrovascular responses between sexes over a wide range of exercise intensities. We demonstrated females were able to maintain MCAv in response to GXT by sustaining a higher CVCi, whereas males maintained similar MCAv values with significant increases in CPP. Although the EtCO_2_ values were similar throughout the GXT, it does appear females experience a greater MCAv response to changes in EtCO_2_ compared to males. This indicates possible mechanistic differences in cerebrovascular control between males and females. Potential mechanisms responsible for these differences include flow‐mediated dilation, hormone concentrations, sympathetic outflow, and many others (Barnes, 2017; Fujii et al., 2020; Jarvis et al., 2011; Kellawan et al., 2015; Peltonen et al., 2015). However, speculation of the individual contribution of these mechanisms would be inappropriate as research in this area is severely lacking. Ultimately, this study opens the door for future research into identifying the mechanisms for cerebrovascular responses, sex‐specific differences in those mechanisms, while also reiterating the importance of inclusion, and separation of female participants.

## DISCLOSURE

The authors declare no conflicts of interest.

## AUTHOR CONTRIBUTIONS

JDA performed data collection, analysis, and drafted the manuscript. DJL and RDL developed research design, data analysis, and edited the manuscript. JHS, JS, JS, JAT, LDA, and AY, performed data collection and edited the manuscript. JMK developed research design, performed data collection, oversaw data analysis, research compliance, and drafted and edited the manuscript.

## Supporting information



Table S1
