# Supplementary material for: Cerebrovascular responses to graded exercise in young healthy males and females
Source: Physiol Rep. 2020 Oct 28;8(20):e14622. doi: 10.14814/phy2.14622 (PMC7592493; doi:10.14814/phy2.14622)
Supplement: Supplementary file 1 — Table S1 [file PHY2-8-e14622-s001.docx]

| Supplemental Table 1. *Change in Cerebrovascular Variables from Baseline During Common Workloads of the GxT in Healthy Young Males and Females* | | | | | | | |
| --- | --- | --- | --- | --- | --- | --- | --- |
|  | **50W** | | **75W** | | **100W** | |  |
|  | Males | Females | Males | Females | Males | Females | *p* value |
| ΔMCAv (cm/s) | 11.13 (4.28) | 15.68 (7.55) | 12.69 (6.22) | 17.37 (7.92) | 15.43 (6.39) | 19.37 (8.84) | 0.169 |
| ΔCPP (mmHg) | 10.59 (7.80) | 15.07 (6.62) | 16.27 (11.00) | 20.62 (6.26) | 21.41 (11.73) | 24.98 (8.01) | 0.293 |
| ΔCVCi  (cm/s/100 mmHg) | 1.21 (7.06) | 0.40 (11.81) | -2.91 (8.96) | -4.07 (13.16) | -5.17 (10.94) | -3.53 (12.48) | 0.957 |
